# Research on New Methods of Topic Mining and Topic Prediction for Medical Preprints on Emerging Infectious Diseases

**DOI:** 10.7759/cureus.85773

**Published:** 2025-06-11

**Authors:** Zongjing Liang, Yun Kuang, Gongcheng Liang, Zhijie Li, Mingfeng Jiang

**Affiliations:** 1 School of Economics and Management, Guangxi Normal University, Guilin, CHN; 2 Library, Guilin Normal University, Guilin, CHN; 3 Network and Educational Technology Center, Guilin Normal University, Guilin, CHN; 4 Institute of Library and Information Studies, Guangxi Normal University, Guilin, CHN

**Keywords:** google trends, information science, lda topic model, medical informatics, preprint research, research topic prediction

## Abstract

Background and purpose

To cope with the continuous risk of sudden infectious diseases and achieve real-time monitoring of research trends, this paper proposes a new prediction framework that combines public attention indicators with medical preprint topic analysis. In view of the lag problem of traditional topic prediction methods, this paper introduces Google Trends data to improve the timeliness of prediction.

Methods

In this study, 18,060 COVID-19-related preprint abstracts were obtained from the medRxiv platform using web crawler technology. The unsupervised probabilistic modeling method, Latent Dirichlet Allocation (LDA), was used to extract the latent topic structure in the text. In order to analyze the dynamic relationship between research topic intensity and public attention, the Autoregressive Distributed Lag (ARDL) model, which can simultaneously process I(0) and I(1) time series, was introduced. Text data preprocessing included word segmentation, stop word removal, lemmatization, and synonym standardization. Time series data were aggregated by week, the original data were logarithmized, the Augmented Dickey-Fuller (ADF) unit root test was used to determine stationarity, and non-stationary variables were differenced. The models were implemented in Python and EViews10, respectively.

Results

Seven major research topics were identified through LDA modeling. ARDL analysis verified that there was a significant dynamic relationship between public search trends and topic intensity, and that the model had good predictive performance.

Conclusion

This study combined LDA with ARDL models to construct a real-time prediction method that can be used to track the evolution of medical preprint topics. This method has important theoretical and practical significance in the field of public health informatics and provides feasible predictive support for the monitoring and prevention of future infectious diseases.

## Introduction

Although the World Health Organization (WHO) has declared the COVID-19 public health emergency to be over, various infectious diseases remain prevalent globally. Real-time prediction of infectious disease research topics can provide objective references for the global response to future outbreaks.

The COVID-19 pandemic, which began in 2020, significantly affected global society, economy, and culture. To control its spread, many countries implemented strict social measures such as home isolation and travel restrictions. These lockdowns effectively curbed transmission but also caused issues like economic stagnation and disruptions to social order. COVID-19, caused by a novel coronavirus, presented unknown pathology and transmission mechanisms, prompting researchers worldwide to urgently investigate its features.

Given the scale of the pandemic, no single entity could manage it independently. International cooperation became vital. Traditional publishing methods - often requiring over six months from submission to publication - were too slow for the fast-moving pandemic, which demanded rapid updates on pathology and vaccine development.

Preprint platforms emerged as a timely solution, allowing researchers to share manuscripts before peer review [1]. This accelerated the exchange of information and provided timely data to support clinical and policy decisions. The rapid publication model helped researchers and decision-makers stay updated in a highly dynamic pandemic environment [2]. In order to fully leverage the role of COVID-19 preprints in epidemic prevention and control, as well as policy decision-making, existing studies have demonstrated that applying mathematical models to quantitatively analyze preprint abstracts and papers can facilitate statistical descriptions and predictive research on the pandemic [3-5].

The current state of related research is summarized as follows. The current research status includes three aspects: research on medical preprints, research on topic mining, and research on topic prediction.

Research on medical preprints

The primary source of raw data for preprint papers on COVID-19 research comes from two open platforms focused on medical research: medRxiv and bioRxiv. The research content includes the quantitative relationship between the pandemic and the number of preprints published. The research outcomes comprise both theoretical studies and quantitative analyses.

Theoretical Research

Taneja et al. and van Schalkwyk and Dudek highlighted the importance of rapid dissemination of scientific and medical findings during the COVID-19 pandemic [1,6]. Huang and Tian analyzed the lead-lag effects between different types of papers over time, which is of significant value for evaluating research [4].

Quantitative Analysis

This includes correlation and regression analyses. For example, Gordon et al. [7] conducted predictive research on COVID-19 papers published on preprint servers like medRxiv and bioRxiv. Älgå et al. [8] pointed out the rapid growth in the number of pandemic research papers published on medRxiv. Älgå et al. [9] mapped the knowledge evolution during the early stages of the COVID-19 pandemic. In summary, current research on COVID-19 preprints mainly focuses on the importance of preprints and quantitative analyses of their correlations.

Research on topic mining

Current topic mining in COVID-19-related papers primarily uses the LDA (Latent Dirichlet Allocation) model.

Zhao et al. used journal papers as the research object and applied a probabilistic topic model method to generate the annual trend of topic strength [10]. Qiu and Shen revealed the research hot topics in the field of big data in China [11]. Li et al. used text-mining techniques to accurately extract spatial and topic information from the metadata of COVID-19-related papers [12]. In summary, current LDA topic mining research mainly focuses on already-published journal papers and social media posts.

Research on topic prediction

Topic prediction research, based on the evolutionary curves derived from LDA modeling, provides valuable references. Existing research primarily uses two types of models for prediction: ARIMA (AutoRegressive Integrated Moving Average) or ARMA models and machine learning models.

ARIMA/ARMA Models

Research outcomes include works such as Yue et al.'s study, which used an ARIMA model to conduct a predictive analysis of major topics in the field of information science [13]. Singhal et al. applied the ARIMA model for sentiment prediction [14].

Machine Learning Models

Dong et al. applied a state-space model to study the relationship between healthcare-related publications and social media [5]. Xu et al. proposed a multi-LSTM (Long Short-Term Memory) model to evaluate the scientific impact of publications [15].

In summary, topic prediction models mainly focus on univariate ARMA or ARIMA models, with a few studies using machine learning models (such as state-space transformation models). These models generally lack the functionality to analyze the lag effect of variables. Currently, there is limited research on LDA topic mining for preprint abstracts or papers. However, LDA topic mining and prediction for preprints, which have the advantage of rapid publication, have significant practical significance for effectively and timely responding to pandemic-related knowledge dissemination.

A review of the current state of domestic and international research reveals that existing LDA-based topic modeling studies primarily focus on published journal articles and social media content, such as Weibo posts. Topic prediction models in these studies are mostly limited to univariate approaches like ARMA and ARIMA models, which lack the capacity to analyze variable lags. At present, there is a notable scarcity of research applying LDA topic modeling to preprint abstracts or full texts. However, given the rapid dissemination advantage of preprints, topic mining and prediction based on such data are of significant practical value for the timely and effective dissemination of epidemic-related knowledge.

To address these gaps, this study selects COVID-19 preprint abstracts as the primary research corpus for conducting LDA-based topic modeling and visualization of classified topics. Furthermore, to capture the correlation and lag effects between topic evolution and public attention during prediction, this study introduces a proxy indicator of public attention - Google Trends. A bivariate Autoregressive Distributed Lag (ARDL) model is constructed to address lag issues between variables and improve the accuracy of COVID-19 topic prediction. The ultimate goal is to provide objective and real-time reference data for the prevention, management, and monitoring of COVID-19 transmission dynamics.

This study combines LDA, ARDL, and Google Trends to achieve the following innovations: (1) Data Integration: for the first time integrating micro-level topic data with macro-level public attention data (Google Trends); (2) Methodological Innovation: proposing a novel combined model of “LDA + ARDL” for predicting topic evolution; (3) Problem Solving: by introducing real-time search indices to address the limitations of traditional topic prediction methods in terms of real-time forecasting and lag issues; and (4) Application Expansion: focusing on the prediction of preprint research related to emerging infectious diseases (e.g., COVID-19), thereby extending the application scope of public attention data (Google Trends) and providing a reference for the effective prevention and control of infectious diseases.

The following sections will elaborate on the research methods and data acquisition, topic evolution research, topic prediction analysis, and conclusions.

## Materials and methods

Dataset

The dataset in this article consists of two parts: Medical Preprint Data and Google Trends Data. (1) Medical Preprint Data: COVID-19 was used as the search keyword to extract data from the medRxiv database, covering the period from March 1, 2020, to February 27, 2022. A total of 18,060 related paper titles, abstracts, keywords, and other information were extracted, forming the raw data for text-mining research. The database can be accessed at medRxiv (https://www.medrxiv.org/). (2) Google Trends Data Collection: Google Trends data was obtained from the Google Trends website (https://trends.google.com/trends). In this study, two sets of Google Trends data were collected: one for the keyword "COVID-19," named "Google trends_1," and the other for the keyword "Vaccine," named "Google trends_2." The data collection scope was global, covering the period from March 1, 2020, to February 27, 2022.

Methods

The research method involves using the LDA model for topic mining and applying the ARDL model for topic prediction. Specifically, the process starts by extracting COVID-19 paper abstracts from the medRxiv preprint platform, followed by data cleaning. A Python program is then developed to implement the LDA topic extraction function, generate relevant graphs, and output the calculated results. Subsequently, the ARDL model is used for topic prediction, and an analysis of prediction accuracy is conducted. Finally, conclusions are drawn. The research method covers concepts and theories related to LDA topics, the ARDL model, and public attention.

LDA model

LDA is an unsupervised probabilistic topic modeling method proposed by Blei et al. in 2003 [16]. The model assumes that each document is composed of a mixture of multiple latent topics, and each topic is represented by a probability distribution of words. LDA discovers the hidden topic structure in the corpus by modeling the joint distribution of words, topics, and documents [17].

In this study, the LDA model is used to identify the main research topics from the abstracts of preprints related to emerging infectious diseases. Each abstract is regarded as a document, and the model outputs the topic distribution of each document and the word distribution of each topic, which helps analyze the evolution trend of research focus over time [18].

The process of solving the LDA model using the Python language: (1) Reading data: for example, reading raw data from an Excel file. (2) Data preprocessing: this process includes using Jieba for word segmentation, removing stop words, replacing synonyms, deleting unnecessary spaces, etc. (3) LDA modeling: this part of the operation includes word frequency vectorization processing, calculating the perplexity under different numbers of topics, finding the optimal number of topics, modeling the optimal number of topics, outputting the results, and outputting various drawings obtained after LDA calculation.

ARDL model

Since the ARDL model was proposed by Pesaran and Shin in 1995, it has been widely used to analyze the short-term dynamic relationship and long-term cointegration relationship between variables [19]. Compared with the traditional cointegration model, the ARDL method can be used for time series data that contain both I(0) and I(1), and does not require the variables to have the same order [20].

According to the research of Liang and Kuang, the ARDL model has the following advantages [21]: (1) the ARDL bound cointegration test can be effectively applied to finite or small sample data series; (2) once the lag order is determined, the OLS (Ordinary Least Squares) regression analysis can be used for a bound test; (3) ARDL can perform cointegration analysis regardless of whether the data series is stationary at the level (I(0)) or the first difference (I(1)); (4) the ARDL model can clearly identify the influencing variables in the regression results; (5) in addition to long-term cointegration analysis, short-term correlation analysis can also be performed.

The model solution steps are as follows: (1) unify the variables involved in the calculation into the same time frequency data; (2) use the Augmented Dickey-Fuller (ADF) unit root test to determine the stationarity of each variable; (3) perform first-order difference processing on non-stationary variables; (4) determine the optimal lag order based on the Akaike Information Criterion (AIC); and (5) estimate the ARDL model and determine whether there is a cointegration relationship between the variables through boundary tests. Model construction and analysis were completed in EViews 10.

This study uses the ARDL model to analyze the dynamic relationship between topic intensity (extracted by LDA) and the public attention index (Google Trends). The model can not only reveal the short-term response mechanism, but also capture the long-term equilibrium relationship.

Reasons for this study choosing the ARDL model: (1) Flexibility with integration order: Unlike conventional regression models (including those with lagged terms), which require all variables to be integrated of the same order, the ARDL model can accommodate regressors that are a mix of I(0) and I(1). This makes ARDL especially suitable for our dataset, as Google Trends search indices are typically very stable (I(0)) or can become stationary after first differencing (I(1)). The topic variable, whether I(0) or I(1), also meets this requirement. (2) Flexible lag structure: In our study, the dependent variable is topic frequency, and the explanatory variable is the public attention index from Google Trends. Existing research shows a time lag between public attention and topic emergence. The ARDL model allows for flexible specification of lag orders, enabling us to identify optimal lag lengths for each topic - an advantage that many machine learning or other time series models lack. (3) Efficient and automated computation: We used EViews 10, which offers a dedicated ARDL module. This module automatically determines the optimal lag structure based on statistical and theoretical criteria, greatly improving computational efficiency and prediction accuracy. Our empirical results also confirmed the model’s high predictive performance.

In this study, Google Trends variables are incorporated into the topic change prediction model. The ARDL model is employed to address the time-lag issue between topic changes and public attention, thus improving the accuracy of predictions.

Public attention

Before the advent of network search indices, the number of traditional media reports was often used as a proxy for public attention. After the release of search indices, many studies began using network search indices as a substitute variable for public attention. A network search index is presented as an index by search companies, reflecting users' search histories. The current representative search indices are the Chinese search index and the English search index, published by Baidu and Google, respectively, and known as Baidu Index and Google Trends. Search indices have the advantages of a large sample size, good real-time performance, and free access. They have been widely used in fields such as society, economy, and medicine. Existing applications include using Google Trends data as a proxy for public demand [22], and Google Trends has been proven to be a suitable source for global public demand survey data [23]. During the COVID-19 pandemic, Google Trends data was used to monitor public feedback on non-pharmaceutical intervention measures implemented by various countries to effectively control virus transmission [24]. In medical publication prediction, Google Trends is used as an indicator of social interest in publications [5]. In this study, Google Trends data will be combined with the ARDL model and co-modeled with time series data of various topics to ultimately improve the accuracy of predictions.

Construction and Validation of the Public Attention Index

The public attention index was constructed using weekly Google Trends data for the keywords “covid-19” and “vaccine,” covering the global region from March 1, 2020, to February 27, 2022. The data were normalized on a 0-100 scale by Google. To correct for potential heteroskedasticity, this study applied a natural logarithmic transformation to the search index. For validation, this study plotted line graphs pairing the public attention index with each topic. The visual inspection indicates a similar pattern of variation between the two. As there exists a time lag between search trends and topic prevalence, the use of comparative trend visualization is appropriate for capturing their dynamic relationship.

## Results

Research on topic evolution

Topic extraction was conducted using a Python program. The program workflow involved reading the data first, followed by data preprocessing, which included standardizing word cases, performing word segmentation using Jieba, removing stop words, replacing synonyms, and deleting blank spaces after preprocessing. Next, LDA model topic extraction was performed. The topic extraction process mainly involved vectorizing word frequency, calculating the perplexity for different numbers of topics, determining the optimal number of topics, and finally, outputting the corresponding Excel data and graphs.

Topic mining

**Figure 1:**
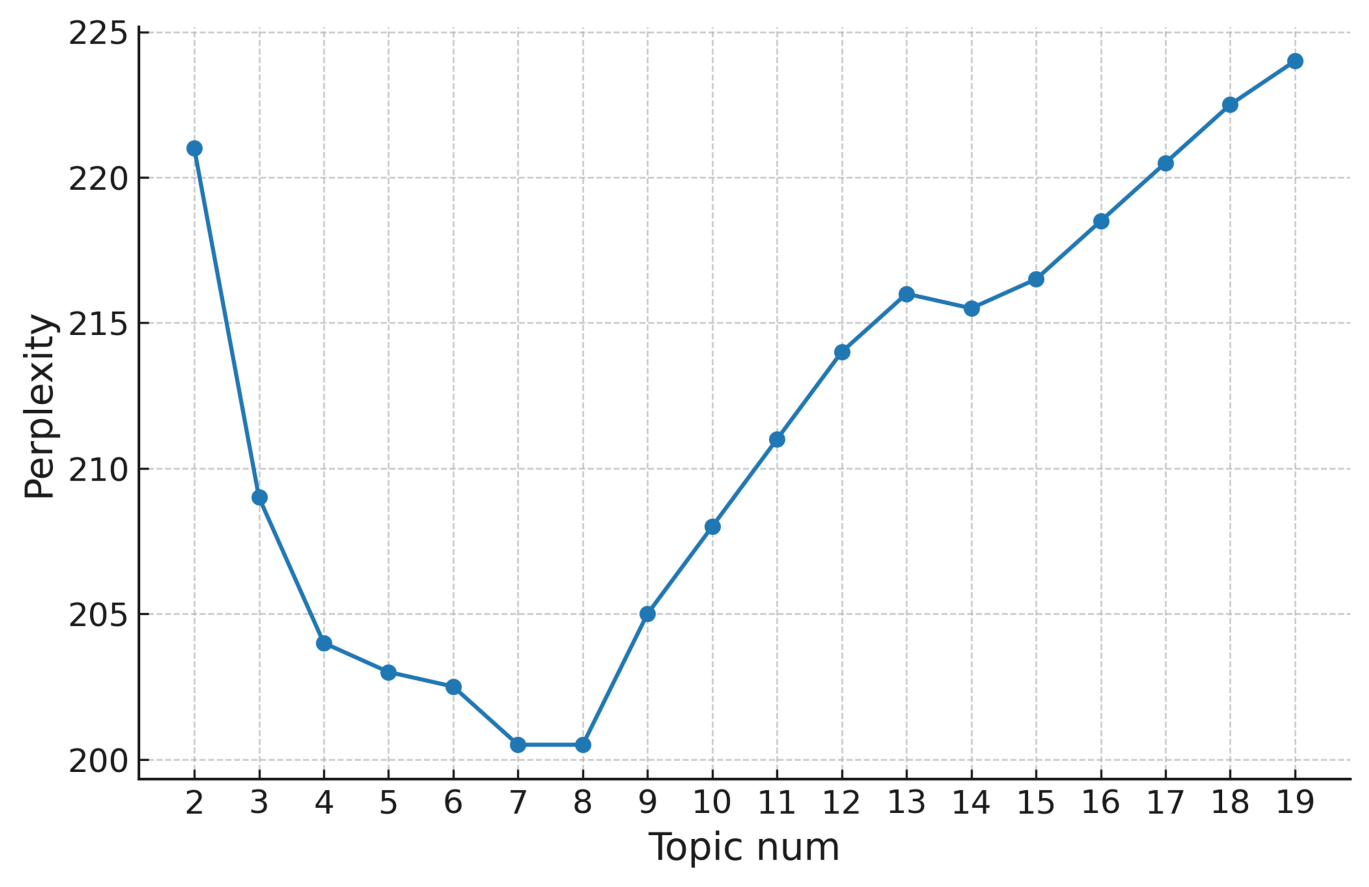
Graph of the Relationship Between Perplexity and Number of Topics (Topic num)

The relationship between perplexity (a measure of model performance) and the number of topics was calculated, and the results are shown in Figure 1.

Based on the principles of LDA construction, as the number of topics increases, the turning point in perplexity indicates the optimal number of topics. As seen in Figure 1, the perplexity is minimized when the number of topics is 7, making 7 the optimal number of topics.

Topic clustering

The programming calculations yielded the topic clusters and the probability of occurrence of the main associated words. The results are presented in Table 1.

**Table 1:** Topic Cluster With the Top Six Keywords and Their Probabilities

Topic Cluster Number	Topic Cluster Name	Six Related Words in Research Text and Their Probabilities					
1	Mental Health Research	Health	Participant	Risk	Age	Symptom	Mental
		0.0138	0.0105	0.0089	0.0073	0.0053	0.0047
2	Vaccine Research	Variant	Vaccine	Sars-cov-2	Vaccination	Infection	Mutation
		0.0308	0.0245	0.0208	0.0179	0.017	0.0093
3	Epidemiology Research	Case	Population	Disease	Prevalence	Expenditure	Malaria
		0.025	0.0139	0.0097	0.0092	0.0089	0.008
4	Complication Research	Patient	Clinical	Disease	Outcome	Risk	Cancer
		0.0411	0.0143	0.0081	0.0078	0.0065	0.006
5	Clinical Epidemiological Diagnosis Research	Care	Mortality	Health	Data	Patient	Death
		0.0178	0.0168	0.0165	0.0131	0.0108	0.0091
6	Modeling Research	Model	Data	Number	Transmission	Case	Analysis
		0.0114	0.0098	0.0074	0.0065	0.0064	0.0034
7	Coronavirus Research	Sars-cov-2	Antibody	Patient	Test	Viral	Response
		0.0206	0.0095	0.0091	0.0086	0.0084	0.0083

The following is a detailed explanation of each topic cluster.

Topic 1: Mental Health Research

Topic Cluster 1 can be summarized as Mental Health Research. Since the outbreak and spread of the COVID-19 pandemic, countries around the world have implemented various response measures, such as school closures, bans on gatherings, and home isolation. These stringent social control measures have undoubtedly had a significant impact on people's mental health. The WHO has compiled global data showing that the COVID-19 pandemic has led to a 25% increase in the prevalence of anxiety and depression worldwide. The WHO also pointed out that women and young people are particularly susceptible to mental health issues. Specific cases in the preprint literature include: (1) a quantitative study using questionnaires to explore the relationship between depressive symptoms, anxiety symptoms, and mental health during pandemic control in several developed countries (e.g., the UK and Italy), and to analyze the impact of lockdowns on lifestyle changes such as sleep, exercise, and social interactions; (2) research on the relationship between eating disorders and mental conditions such as self-harm during the pandemic, which found that self-harm and eating disorders are closely related to depressive and anxiety symptoms during the pandemic.

Topic 2: Vaccine Research

Topic Cluster 2 can be summarized as Vaccine Research. The development and distribution of COVID-19 vaccines are crucial for saving lives and protecting people from severe illness and disease. Vaccines help the body develop immunity to suppress viral growth. Given that the coronavirus, as a severe infectious disease, causes significant harm to the human body, vaccines are essential for ensuring health. Typical cases in preprints include: (1) research evaluating the impact of government mandates for vaccination when accessing public places and non-essential businesses; (2) studies assessing the effectiveness and safety of vaccine development [25]; (3) analysis of the relationship between countries' GDPs and the progress of COVID-19 vaccination from a global perspective. This topic outlines the importance and urgency of effective vaccines for the novel coronavirus, emphasizing that the virus has caused enormous losses in terms of the economy, health, and lives.

Topic 3: Epidemiology Research

Topic Cluster 3 can be summarized as Epidemiology Research. One of the epidemiological characteristics of COVID-19 is the rapid increase in clustered cases. There is an urgent need for global monitoring to detect and potentially predict sources of high pathogenicity, facilitating interdisciplinary scientific communication, data sharing, and coordinated efforts to prevent future pandemics. Typical cases in the preprints include: (1) using electronic health records for epidemiological research; (2) research on causal models of pathophysiological processes based on disease clinical presentations, focusing on their application in predicting the COVID-19 pandemic; (3) a study using the characteristics of the COVID-19 pandemic in Australia as an example to illustrate how monitoring the pandemic helps understand the dynamics of its spread; (4) theoretical research on the early implementation of non-pharmaceutical interventions; (5) research on the community transmission patterns of infectious diseases.

Topic 4: Complication Research

Topic Cluster 4 can be summarized as Complication Research. Studies have shown that patients with comorbidities, including cancer, are at higher risk of death or of developing more severe forms of COVID-19. Cancer progression, recurrence, and metastasis may be related to the coronavirus, suggesting that cancer is one of the complications of COVID-19. Typical cases in preprints include: (1) research showing that the COVID-19 pandemic has led to cardiovascular diseases, emphasizing the need for early detection of complications to improve patient outcomes; (2) studies discovering possible causes of complications, suggesting that COVID-19 patients exhibit different clinical complication characteristics, with genetic and immune backgrounds potentially influencing viral load and host response, contributing to complications; (3) research on the differences in treatment outcomes across different racial genes; (4) studies on the association of COVID-19 with various neurological complications, including stroke, delirium, and encephalitis, which may also trigger psychiatric symptoms.

Topic 5: Clinical Diagnosis Research

Topic Cluster 5 can be summarized as Clinical Diagnosis Research. In the early stages of the COVID-19 pandemic, a significant number of medical preprints focused on the design, development, validation, and implementation of diagnostic testing protocols. These explorations provided valuable attempts for future efforts to effectively control virus transmission. Typical cases in preprints include: (1) identifying croup as one of the main clinical variants of the coronavirus, with clinical diagnosis showing symptoms such as stridor, hoarseness, barking cough, and varying degrees of respiratory distress following infection; (2) research on COVID-19 screening systems, indicating that ocular manifestations are often associated with COVID-19 patients; (3) proposing that rapid antigen diagnostic tests could serve as an alternative method for diagnosing COVID-19; (4) identifying major factors that hinder early diagnosis of the disease; (5) emphasizing research on the effects of clinical viral infection, highlighting the importance of early and accurate clinical diagnosis in managing viral infections.

Topic 6: Epidemiological Modeling Research

Topic Cluster 6 can be summarized as Epidemiological Modeling Research. The necessity of establishing COVID-19 prediction models has become a core task in controlling the pandemic, as modeling can provide early predictions and information, giving policymakers valuable lead time. Typical cases in preprints include: (1) using the SEIR model and Bayesian inference framework to estimate the number of infections in Africa; (2) evaluating the transmission patterns of COVID-19 using classic models (CMs); (3) developing a COVID-19 prediction model using a support vector regression model combined with a random forest algorithm; (4) researching the correlation between pedestrian footprints and virus transmission using a related model; (5) highlighting the extensive application of big data in epidemic modeling. Types of big data, such as network search indices (Baidu Index and Google Trends) and social media data, are integrated into the modeling process to improve prediction accuracy.

Topic 7: Coronavirus Research

Topic Cluster 7 can be summarized as Coronavirus Research. COVID-19 is a variant of the coronavirus, with the core virus still being a coronavirus. Its resurgence could be persistent, and this topic primarily focuses on the evolutionary history of coronaviruses, providing historical data references for current COVID-19 prevention and control. Typical cases in this preprint topic include: (1) reviewing and reconstructing the background and history of research in the field of coronaviruses; (2) clarifying the basic knowledge of coronavirus virology; (3) emphasizing the potential for coronavirus outbreaks to become long-term endemic diseases, which would play a crucial role in public health policy-making; (4) summarizing the seasonal patterns of coronavirus outbreaks; (5) conducting comparative studies on the virulence of different coronaviruses, using infection models to compare the severity of past viruses, and providing references for current pandemic prevention efforts.

Topic visualization

**Figure 2:**
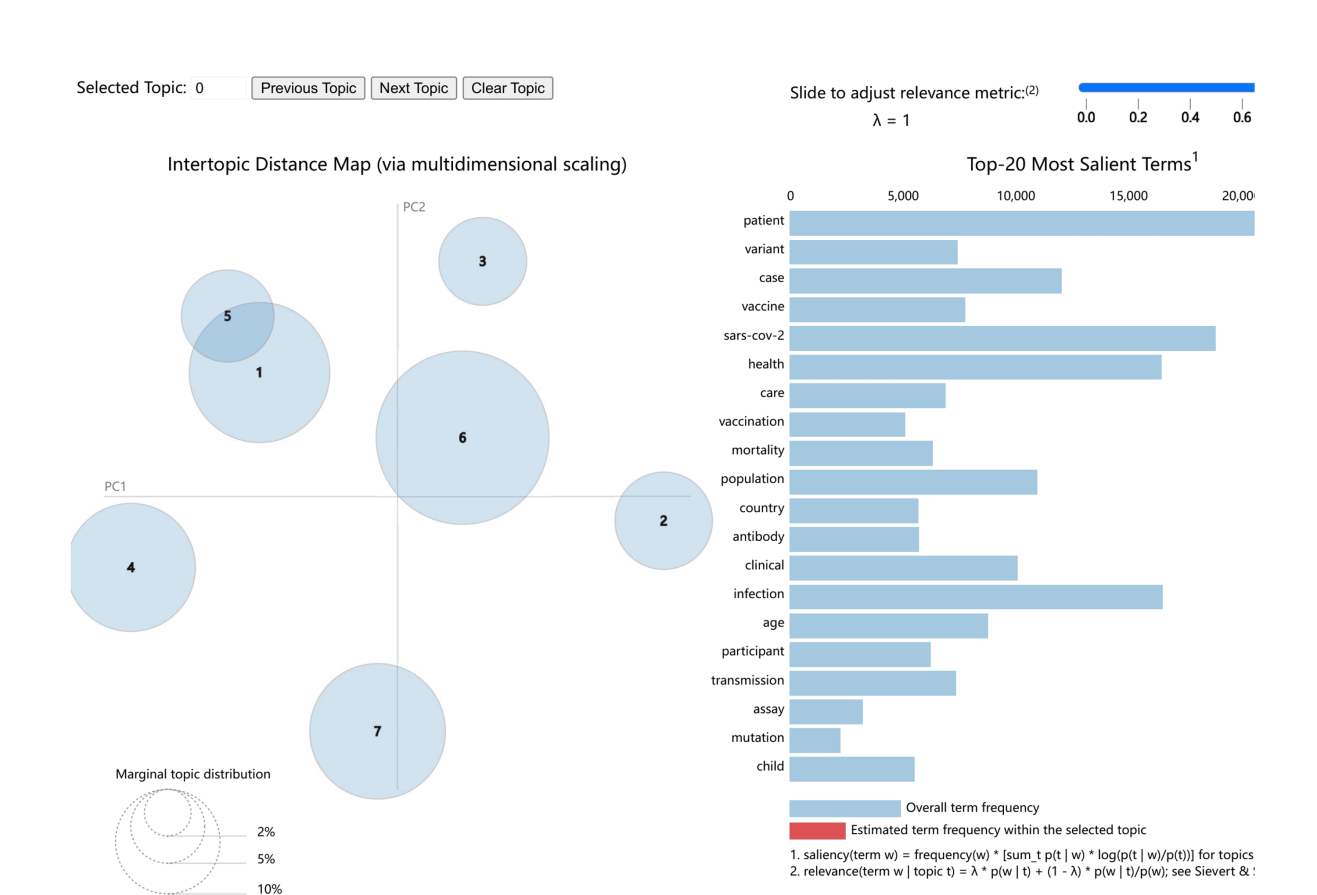
Topic Bubble Chart

To visually display the relative features of the seven major topics, a bubble chart was constructed using the topic function in Python to model the LDA topics. The calculated results are shown in Figure 2.

Figure 2 is a bubble chart visualizing the seven topics, created using Python's pyLDAvis library. In the figure, the numbers inside the circles on the left represent the topic labels. The diameter of each circle indicates the number of documents associated with that topic - the larger the diameter, the more documents it represents. The distance between circles reflects the relatedness between topics: the closer the circles, the stronger the correlation between those topics, and vice versa. The bar chart on the right displays the top N words ranked by their probability within each topic’s word distribution. As shown in Table 1, Topics 1-7 correspond to: Mental Health, Vaccine Research, Epidemiology, Complication Research, Clinical Diagnosis, Epidemiological Modeling, and Coronavirus Research. From Figure 2, we observe that Topic 6 (Epidemiological Modeling) has the largest circle, indicating it includes the most documents. The next largest circle represents Topic 1 (Mental Health), followed by Topic 7 (Coronavirus Research), suggesting these topics have the second- and third-highest document counts, respectively. The distances between the circles also reveal important relationships. Topic 1 and Topic 5 are the closest, and even partially overlap, indicating a strong correlation between Mental Health Research and Clinical Diagnosis. Similarly, Topic 6 (Epidemiological Modeling) is relatively close to Topic 2 (Vaccine Research) and Topic 3 (Epidemiology). In contrast, Topic 4 (Complication Research) and Topic 7 (Coronavirus Research) are positioned farther away from the other topics, suggesting they are relatively independent and less correlated with the others.

Topic evolution analysis

**Figure 3:**
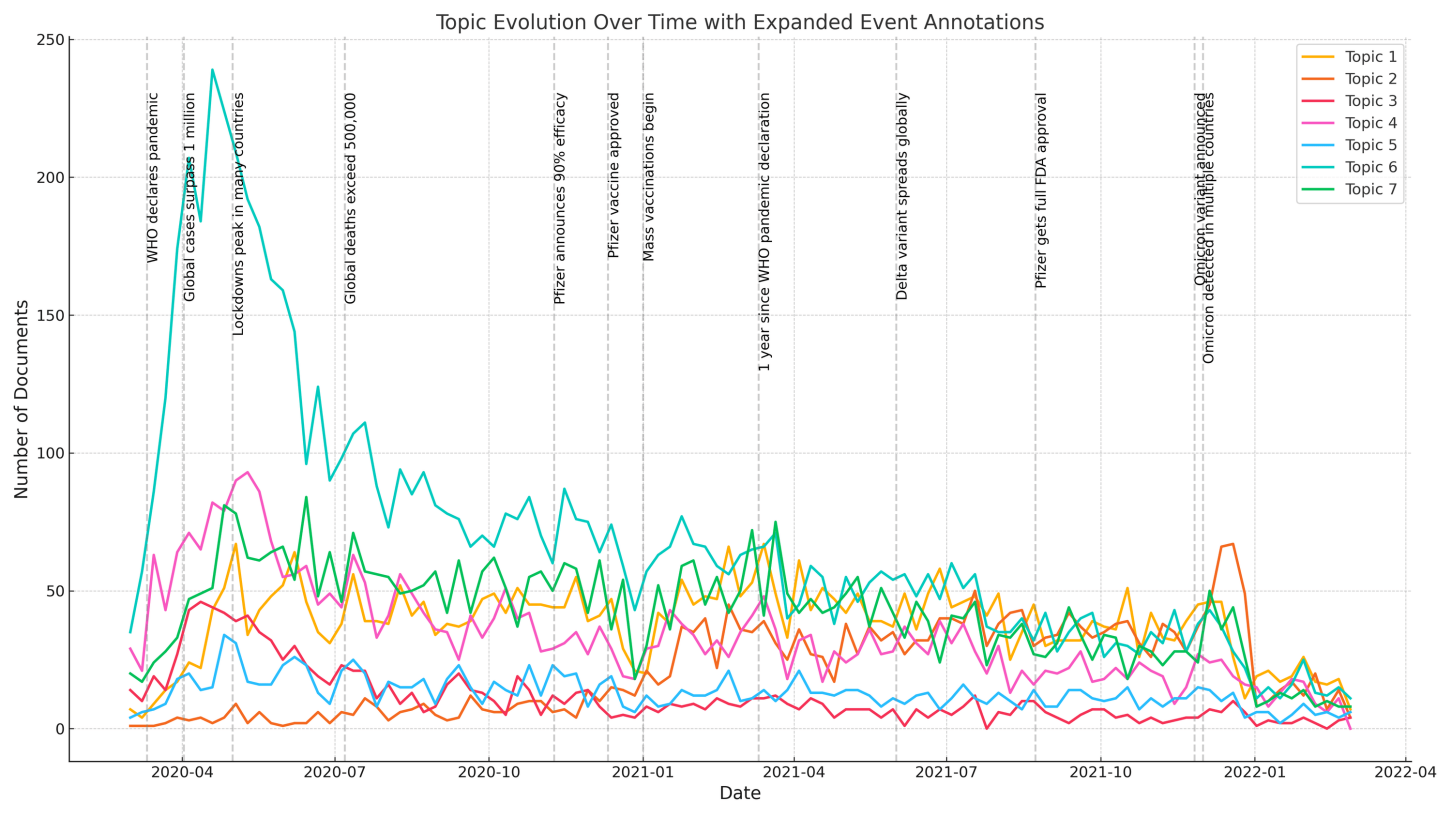
Time Evolution Curves of the Seven Major Topics

To facilitate the observation of the time series evolution of the seven major topics and provide preliminary knowledge for subsequent topic prediction, time series evolution charts for each topic were generated based on the calculated results. Figure 3 shows the time evolution curves of the seven main research topics extracted by the LDA model. The horizontal axis represents the publication date of each week (from January 2020 to February 2022), and the vertical axis represents the number of documents belonging to the topic each week.

This paper summarizes the topic intensity (i.e., the proportion of a certain topic in the document) in each document by week, constructs a time series for each topic, and thus dynamically tracks the changes in research interests. The main findings include: (1) the dominance of modeling research (Topic 6): Topic 6 corresponds to epidemic modeling and prediction, which showed the highest peak and the largest fluctuation in early 2020, indicating that it was the most active research field in the early stage of the epidemic; (2) the gradual rise of vaccine research (Topic 2): Topic 2 involves vaccine-related research, and the number of documents has steadily increased over time, which is in sharp contrast to other topics, reflecting the shift of public attention during the advancement of the epidemic; and (3) common trends of other topics: Topics 1, 3, 4, 5, and 7 all show a trend of rapid rise first and then gradual decline, which is in line with the general pattern of the evolution of the life cycle of epidemic research.

Event Description

To enhance the realistic explanatory power of the topic evolution trend chart, this article marks 12 key public health events in Figure 3. These events are arranged in chronological order as follows: On March 11, 2020, the WHO officially announced that COVID-19 constitutes a global pandemic. Around this node, related research topics began to grow rapidly. On April 2, 2020, the global cumulative number of confirmed cases exceeded 1 million, and the global public health response entered a new stage. In early May 2020, countries adopted strict lockdown measures, and the focus of public health research shifted to social impact, mental health, and other aspects.

On July 7, 2020, the global cumulative death toll exceeded 500,000, triggering a shift in research topics to analysis of causes related to death. On November 9, 2020, Pfizer announced the effectiveness of mRNA vaccine prevention and control, triggering a shift in research topics to vaccine-related content. On December 11, 2020, the US FDA granted Pfizer's vaccine emergency use authorization, and the world's epidemic prevention and control entered a new stage. In early January 2021, vaccine efficacy research became a hot topic. On March 11, 2021, the first anniversary of the COVID-19 outbreak, the research topic shifted to policy response research. In early June 2021, the Delta variant appeared and spread, and the corresponding research topic also became a hot topic. On August 23, 2021, the landmark event of this time was the entry of Pfizer's vaccine from the emergency use stage to the routine vaccination stage. On November 26, 2021, the Omicron variant appeared and became a new topic. In early December 2021, the Omicron variant appeared in many countries, causing global discussions on vaccine efficacy.

Topic prediction analysis

In summary, the topic prediction derived from the LDA model has significant applications in various fields, including information management, text-mining, personalized recommendation, and decision support. It not only enhances data processing efficiency but also serves as a powerful tool for the in-depth analysis and understanding of textual data.

By solving for the time series of topic evolution using the LDA model, the trends of each topic can be identified. To track the real-time changes of each topic and provide real-time reference data for understanding future trends, predicting these topics becomes particularly important. Several studies have already been published on topic prediction. For instance, using the ARMA model for LDA topic prediction, the average error in public opinion topic prediction was found to be less than 5.64% [19].

Currently, preprint topic prediction methods primarily use ARMA or ARIMA models. However, both models are limited in their ability to handle lag variables. Existing research indicates that preprints have a correlated relationship with public attention. To overcome the shortcomings of current topic prediction models, which lack consideration of lag characteristics, this section combines public attention variables with topic evolution variables to construct an ARDL model, enabling predictive analysis of topic evolution.

The following research steps will first involve analyzing the raw data, then constructing ARDL models for different topics, and finally, conducting in-sample static predictions to examine the measurement coefficients of the prediction models, demonstrating the effectiveness of using the ARDL model for topic prediction.

Building the ARDL Model

**Figure 4:**
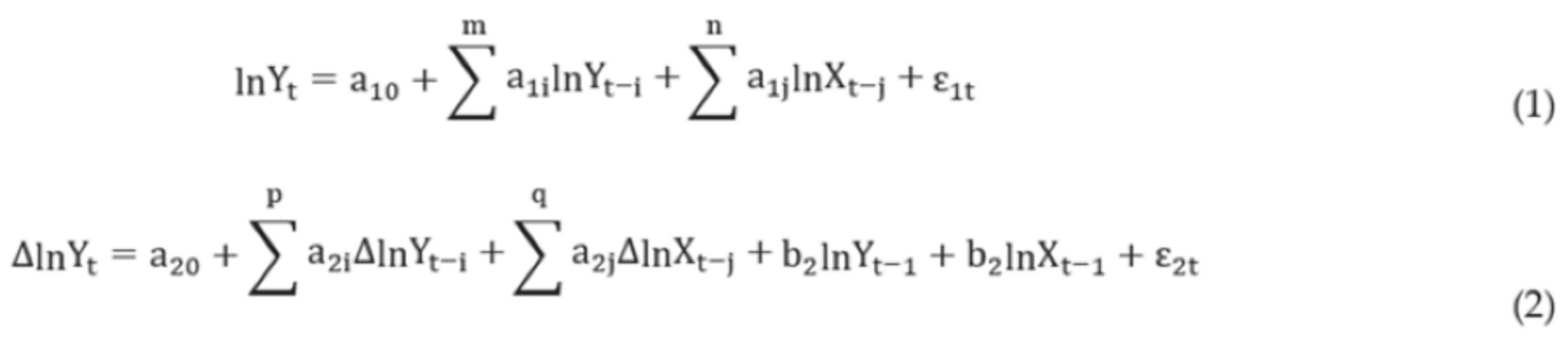
ARDL Model Calculation Formula ARDL, Autoregressive Distributed Lag

According to the modeling requirements of the ARDL model, the specific steps begin with testing the stability of the data series, typically using the ADF test. Next, a boundary test is conducted, followed by the analysis of long-term cointegration relationships and short-term fluctuations. The general form of the ARDL model is shown in Figure 4 [20,26,27].

In Figure 4, Equation 1 represents the level form of the ARDL model, while Equation 2 represents the error correction form (ECM) of the ARDL model. In these equations, t represents time, Yₜ indicates the attention to each LDA preprint topic, Xₜ represents the Google search index, and Yₜ₋ᵢ, Yₜ₋ⱼ, Xₜ₋ᵢ, and Xₜ₋ⱼ represent the lagged terms of Y and X by i and j periods, respectively. Constants a₁₀, a₂₀, and a₃₀ are intercept terms, and ε is the error term. The variables p, q, m, and n indicate the number of lags, and ln and Δ denote logarithms and differencing, respectively.

The steps for parameter estimation in the ARDL model are as follows [19]: (1) Testing for Stationarity: The ARDL (m, n) model requires that the data series used for modeling must be integrated of order 0 or 1 (I(0) or I(1)), but they do not need to be stationary at the same order. (2) Estimating Model Parameters: The key to determining model parameters lies in selecting the lag order for the two variables. The lag order is chosen based on the AIC, selecting the model with the smallest AIC value. (3) Boundary Test and Model Stability Check: This involves calculating the model's F-statistic and comparing it against critical values to assess the model's validity. The stability and accuracy of the model are further evaluated using the CUSUM and CUSUMSQ (Cumulative Sum of Squares) tests [28]. (4) Constructing the Long-Term Cointegration Equation: Once the ARDL model passes all tests, it is used for prediction analysis, and the prediction results are interpreted. The cointegration test in the ARDL model primarily uses the F-statistic to determine whether a long-term cointegration relationship exists between variables. If the F-statistic falls within the critical value bounds for a given significance level, it indicates that a long-term cointegration relationship exists; otherwise, it does not. The reference value of the F-statistic depends on the number of variables, the order of integration, and the error term settings. In practical application, comparing the calculated F-statistic with the corresponding boundary value allows one to judge whether a cointegration relationship exists [19]. Once the ARDL model passes all tests, it can be used for in-sample or out-of-sample forecasting, providing quantitative reference data for policy research.

Raw Data Analysis

**Figure 5:**
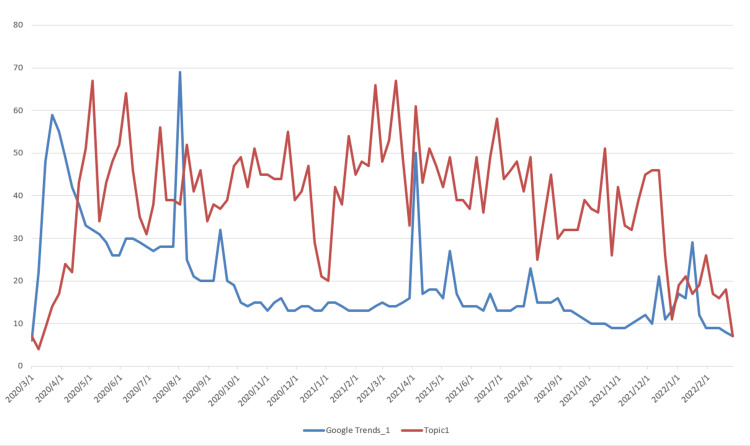
Time Series Plots of Topic 1 Data and Google Trends

**Figure 6:**
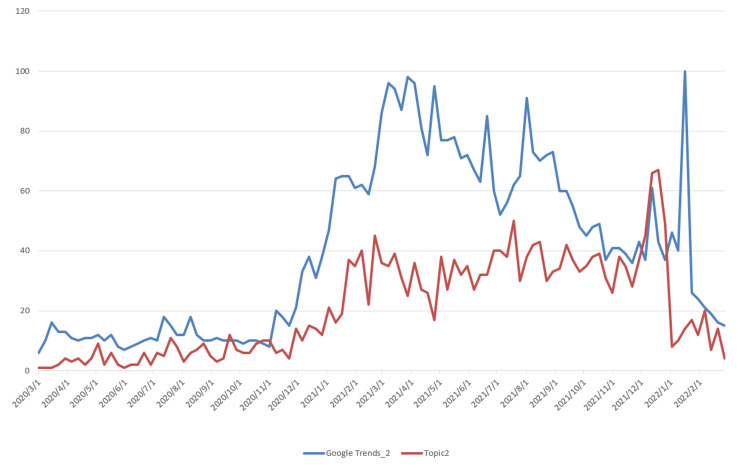
Time Series Plots of Topic 2 Data and Google Trends

**Figure 7:**
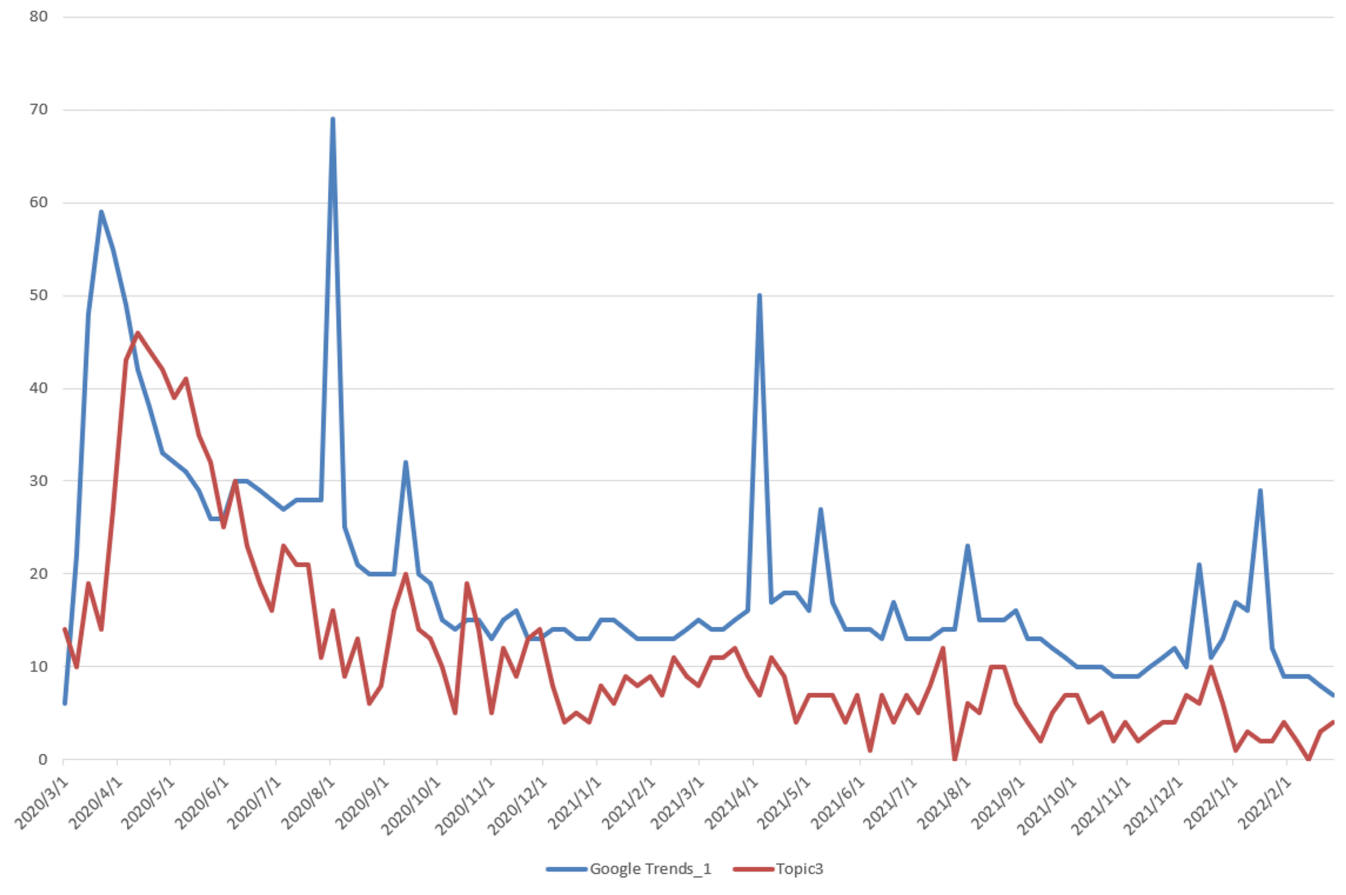
Time Series Plots of Topic 3 Data and Google Trends

**Figure 8:**
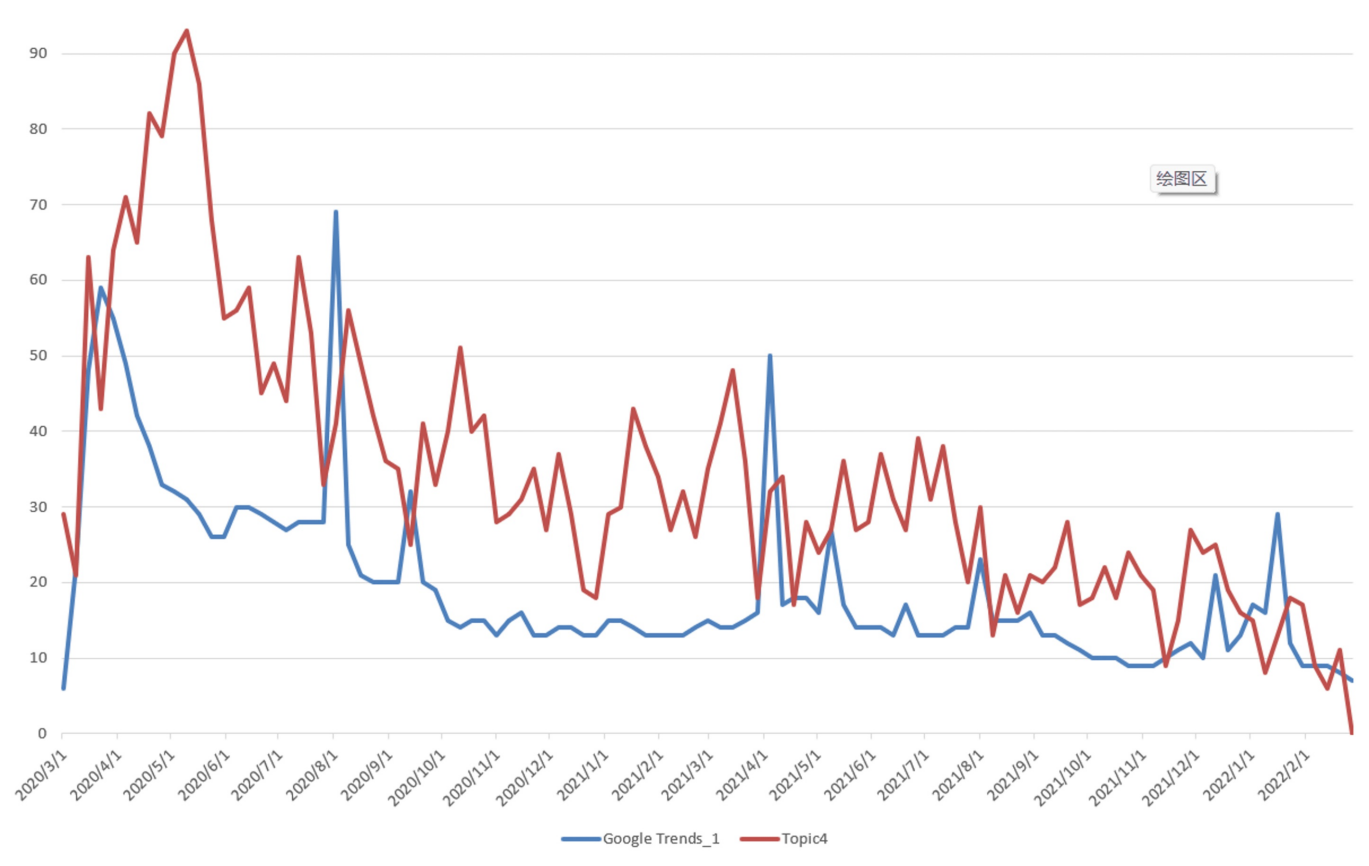
Time Series Plots of Topic 4 Data and Google Trends

**Figure 9:**
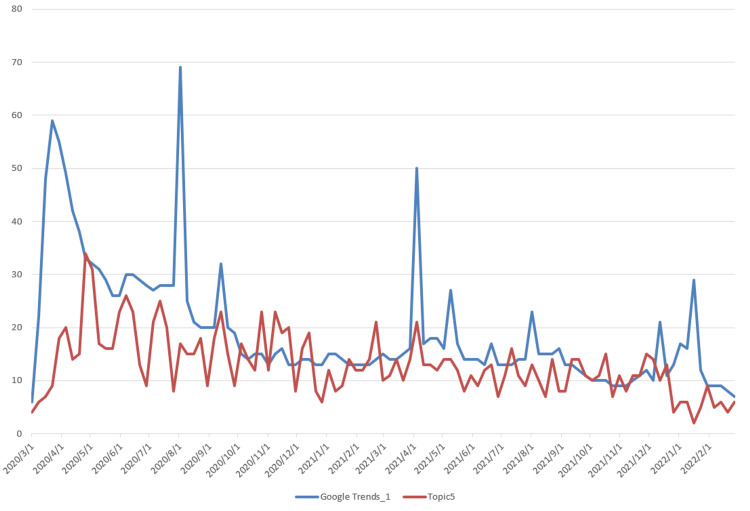
Time Series Plots of Topic 5 Data and Google Trends

**Figure 10:**
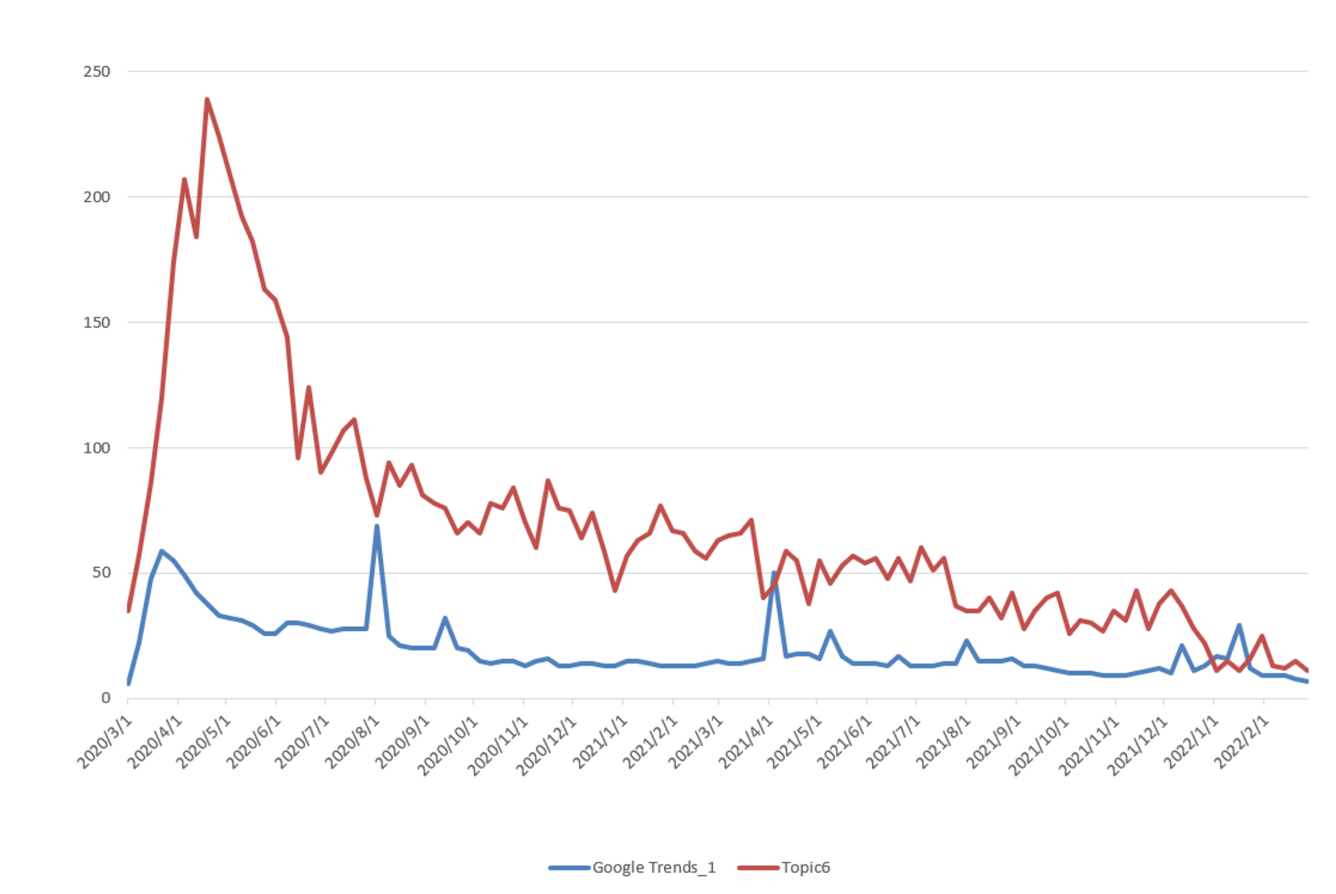
Time Series Plots of Topic 6 Data and Google Trends

**Figure 11:**
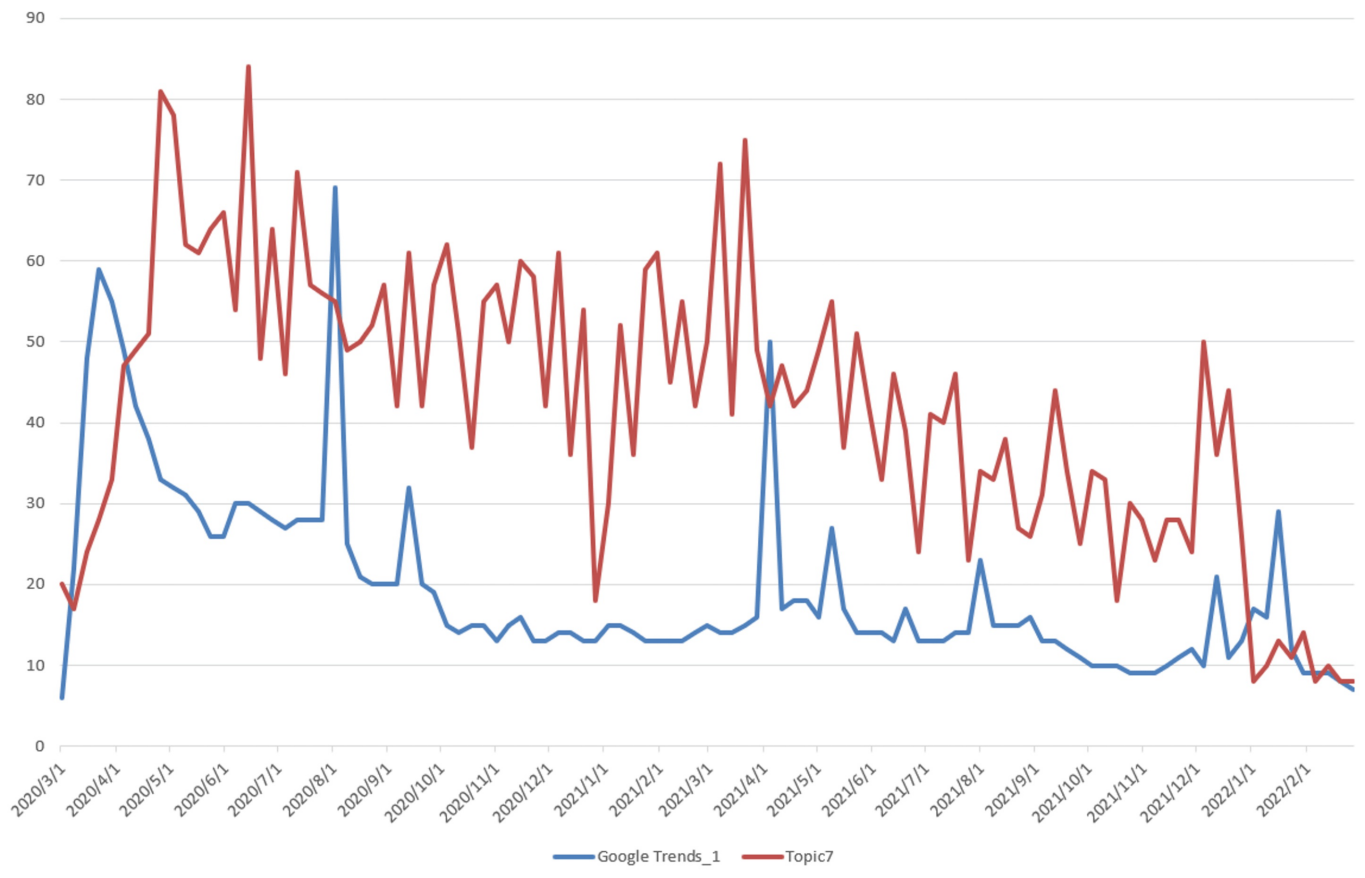
Time Series Plots of Topic 7 Data and Google Trends

The data used for topic prediction modeling consists of two components. The first is the topic data, which was obtained by applying the LDA model. The corresponding time series data are shown in Figure 3. The second component is the Google Trends data, which was retrieved from the official Google Trends website (https://trends.google.com/trends). The observation results indicate that Topic 2, which is related to vaccine research, shows a steadily increasing trend, differing from the other six topics, which exhibit a rapid initial rise followed by a gradual decline. Based on this, we extracted two data series from Google Trends: “Google Trends_1,” corresponding to the search keyword “COVID-19,” and “Google Trends_2,” corresponding to the keyword “vaccine,” with the latter specifically associated with Topic 2. The time series data of Topics 1-7 and their corresponding Google Trends are shown in Figures 5-11.

From Figures 5-11, we can observe that the time series data of Topics 1, 3, and 4-7 exhibit a similar trend to that of Google Trends_1, with values generally decreasing over time. In contrast, the data for Topic 2 (i.e., vaccine research) aligns with Google Trends_2 in showing a gradual increase in values. This pattern reflects the real-world situation, in which public attention to vaccine research grew gradually after the outbreak began, whereas public interest in the other topics followed a declining trend.

According to the modeling requirements of the ARDL model, the stationarity of the original data must first be tested. The ADF test is generally used for this purpose. The results of the ADF unit root test for the topic data and Google Trends, comprising a total of nine columns of data, are shown in Table 2.

**Table 2:** ADF Test for Original Data Series ADF, Augmented Dickey-Fuller

Variable Description	Variable	T-statistic	p-value	Unit Root Order
Google Trends_1	lnX1	-3.1835	0.0238	I (0)
Google Trends_2	lnX2	-15.06	0	I (1)
Topic 1	lnY1	-4.1908	0.0011	I (0)
Topic 2	lnY2	-7.9796	0	I (1)
Topic 3	lnY3	-16.16	0.0001	I (1)
Topic 4	lnY4	-3.023	0.036	I (0)
Topic 5	lnY5	-12.8325	0	I (1)
Topic 6	lnY6	-13.0007	0	I (1)
Topic 7	lnY7	-10.1753	0	I (1)

From Table 2, it can be seen that all data series are either stationary at level (I(0)) or become stationary after first differencing (I(1)). According to the modeling requirements of the ARDL model, the data used for ARDL modeling must be stationary at the 0th or 1st order. Based on the ADF test results in Table 2, it can be concluded that the data series meets the ARDL modeling requirements.

Model Construction

With the data series meeting the prerequisites for ARDL modeling, the specific calculations for each topic are carried out and the corresponding parameters are obtained. The calculation results are shown in Table 3.

**Table 3:** Model Form and Boundary Test Results ARDL, Autoregressive Distributed Lag

Subject Title	ARDL Form	F-statistic	Error Precision	R^2^
Topic 1	ARDL (2, 0)	9.00	1%	0.59
Topic 2	ARDL (5, 2)	4.22	5%	0.83
Topic 3	ARDL (2, 5)	3.34	10%	0.67
Topic 4	ARDL (12, 0)	8.97	1%	0.78
Topic 5	ARDL (7, 6)	7.47	1%	0.71
Topic 6	ARDL (2, 1)	3.24	10%	0.90
Topic 7	ARDL (2, 0)	3.30	5%	0.65

In Table 3, the ARDL form represents the specific ARDL model form derived for each topic after calculation. The first number in parentheses represents the lag order of the topic, and the second number indicates the lag order of the Google Trends data. The F-statistic represents the F-statistic value from the ARDL model's boundary test, and the error precision indicates the margin of error for the F-statistic. According to ARDL theory, when the error precision of the F-statistic is less than 5%, it can be considered that the constructed model has passed the boundary test. The R² in Table 3 represents the fit accuracy of the constructed model. From the results in Table 3, it can be seen that all the constructed ARDL models have passed the boundary test, indicating that there is a cointegration relationship between the model variables. The fit accuracy of the models also shows that most topic models have a good fit. Finally, the ARDL models need to undergo a model stability test, and the test results confirm that all constructed models meet the required precision. This concludes that each topic's ARDL model has met all the theoretical requirements, providing an objective model basis for topic forecasting.

Topic forecasting

To evaluate the in-sample prediction accuracy of the ARDL model, we used three commonly used statistical indicators: root mean square error (RMSE), mean absolute error (MAE), and Theil’s inequality coefficient. These indicators can comprehensively measure the prediction error. The smaller the value, the better the model’s prediction performance [29]. The forecasting results are shown in Table 4.

**Table 4:** In-Sample Prediction Error Evaluation Metrics for Each Topic

Subject Title	Root Mean Square Error (RMSE)	Mean Absolute Error (MAE)	Theil Inequality Coefficient
Topic 1	0.3584	0.2885	0.05
Topic 2	0.4616	0.3636	0.08
Topic 3	0.5440	0.4320	0.12
Topic 4	0.2294	0.1567	0.04
Topic 5	0.2721	0.1906	0.05
Topic 6	0.4033	0.3312	0.05
Topic 7	0.4136	0.3225	0.06

Table 4 shows the prediction error results for the seven topics. As can be seen from the table, the RMSE values of each topic range from 0.2294 to 0.5440, and the MAE values range from 0.1567 to 0.4320, indicating that the overall prediction deviation is small. The Theil coefficient of all topics is less than 0.12, which further verifies the reliability of the prediction.

From the perspective of prediction accuracy ranking, the best performance is seen in Topic 4 (Complication Research), Topic 5 (Clinical Diagnosis), and Topic 1 (Mental Health). Even the relatively high error of Topic 3 (Epidemiology Research) is still within an acceptable range. Overall, the ARDL model achieves stable and reliable short-term prediction effects in multiple topic dimensions.

## Discussion

By summarizing and analyzing the research conclusions of this paper, we can arrive at the following understandings.

Realization of automatic classification of topic importance in COVID-19 research

One of the key findings of this study is the automatic classification of the importance of the topics extracted from medRxiv's COVID-19 preprint publications using the LDA model [16]. The most important topics identified are Topic 6 (Epidemiological Modeling Research), Topic 7 (Coronavirus Research), and Topic 1 (Mental Health Research). Epidemiological modeling, being the most critical topic, aligns with the actual situation, as early prediction of the spread and speed of the disease is essential during the pandemic for allocating resources, health interventions, policy-making, and regional cooperation [24]. Simultaneously, drawing on historical lessons is also crucial for the prevention and control of infectious diseases, making the investigation of COVID-19's origins particularly significant. The WHO has found that, to prevent large-scale transmission, strict quarantine measures were implemented in many countries. While these measures effectively blocked the spread of the virus, they also had a negative impact on the public's mental health, leading to a rise in psychological issues related to mental disorders, which in turn resulted in a surge of preprints on the topic. Therefore, considering the actual circumstances of disease transmission, the classification of topic importance in this study is accurate.

Preprints enable rapid dissemination of health information

Given the rapid spread of the COVID-19 pandemic and its high mortality rate in the early stages, the swift dissemination and sharing of research findings were crucial for controlling the outbreak [6]. The surge in preprints on pandemic research significantly contributed to collaborative efforts to combat the disease, enabling the rapid spread of knowledge. This provided essential information to global scientists, medical professionals, and the public, thereby accelerating the development of related drugs and reducing transmission rates. Although preprints have not undergone peer review and their conclusions require further validation, their publication still represents a valuable research effort. Therefore, it can be considered that preprints offered a rapid information exchange platform for COVID-19 research and strategies, aiding global researchers and the public in responding more quickly and effectively to this global health crisis [8].

Understanding evolutionary trends of medical preprint topics through public attention

Currently, LDA topic prediction methods mainly use the ARIMA model [13]. However, the ARIMA model does not account for lagged variables, whereas the publication of COVID-19 preprints and public attention are characterized by such lags. Introducing public attention indices into the prediction model is therefore a beneficial attempt, and the study results demonstrate that prediction accuracy is improved. In this study, Google Trends - a search index provided by Google based on global search engine users - was used as a proxy for public attention [22]. Google Trends can be widely applied to capture public interest. By using Google Trends as a measure of public attention, this study extends and supplements traditional sources of public attention data, enabling real-time prediction. This approach helps improve the response speed to major infectious disease crises and the real-time management of public opinion, thereby demonstrating the value of Google Trends in empirical research on preprints.

Theoretical contributions to the field of infodemiology

The study’s theoretical contributions are twofold: first, it enriches the field of health informatics, and second, it provides a combined algorithm for empirical research on preprints. COVID-19 preprints have generated a large body of literature in the field of infodemiology. Infodemiology, which emerged with the growing application of network technology and the significant social impact of various infectious diseases, is a combination of information technology and epidemiology [1]. Its theoretical essence is to use big data from the internet to monitor, predict, and respond to epidemics - providing quantitative reference data to support epidemic prevention and control, and to propose targeted policies. The second theoretical contribution of this study is the introduction of a new combined algorithm for empirical preprint research. The research methodology integrates text content mining, the application of search indices, and time series analysis. By combining multiple methods, this study enhances existing methodologies, improves prediction accuracy, and thus provides a new paradigm for empirical research on preprints.

Practical contributions of medical preprints to society

The societal functions of preprints are realized in the following aspects. (1) Public Health Education: The COVID-19 pandemic is a global public safety crisis, and the public, researchers, and social managers need timely information on the spread and control methods of the virus. Preprints can fulfill this role by disseminating public health information [5]. (2) Rapid Publication of Research Findings: While the dissemination of information related to the pandemic and scientific knowledge can occur through social media, preprints enable the rapid global dissemination of professional knowledge, facilitating the sharing of medical research findings. (3) Providing Real-Time Data and References for Management: For government agencies, especially public health authorities, real-time data can assist in formulating more targeted policies, contributing to quantitative, scientific, and precise decision-making [9].

Theoretical and practical value of the LDA-ARDL prediction framework

The LDA-ARDL prediction framework developed in this study has high theoretical significance and application value in the field of public health [30,31]. Based on large-scale medical data and combined with the public search index (Google Trends), the framework can dynamically track the development of research topics related to major infectious diseases, effectively making up for the shortcomings of traditional topic analysis methods in terms of timeliness and predictability. By introducing ARDL time series modeling, not only can the short-term correlation between public attention and research topics be identified, but also their long-term cointegration mechanism can be understood, providing a reliable basis for predicting changes in research hotspots [32,33]. In addition, the model shows good fit and prediction stability within the sample [34]. For public health - especially in the early stages of an epidemic - it is crucial for risk warning and crisis management to attract the attention of the public and academia in a timely manner. Furthermore, the research framework is highly flexible and can be extended to other major public health events (such as monkeypox and dengue fever), thereby providing methodological support and empirical paths for building a more forward-looking global health governance system.

## Conclusions

In order to cope with the continuous risks brought by sudden infectious diseases and to enable real-time monitoring of scientific research trends, this paper proposes a new prediction framework that combines public attention indicators with medical preprint topic analysis. This framework is used to track and predict the evolution of COVID-19-related medical preprint research topics. The paper identifies and analyzes the evolution trends of seven core research topics through 18,060 preprint abstracts collected from the medRxiv platform. The results show that there is a long-term cointegration relationship between each research topic and the corresponding level of public attention. The empirical findings indicate that the prediction accuracy for each topic is strong. This study represents an innovation in research methodology. To our knowledge, this is the first time that micro-level scientific research topics have been combined with macro-level public attention. By integrating text analysis techniques and time series statistical models, the evolution of topics can be effectively tracked and predicted.

This paper provides a useful data reference for the prevention and control of major infectious diseases by studying the evolution and prediction of COVID-19 preprint topics. The research conclusions will help explore the intrinsic value of medical preprints and provide a new approach to quantitative research on preprints. The research methods and conclusions of this article have important theoretical and practical value in the field of public health, and they can provide feasible predictive support for the monitoring and prevention of major infectious disease outbreaks in the future.
